# Impaired Sensory Processing During Low-Oxygen Exposure: A Noninvasive Approach to Detecting Changes in Cognitive States

**DOI:** 10.3389/fpsyt.2020.00012

**Published:** 2020-01-31

**Authors:** Todd R. Seech, Matthew E. Funke, Richard F. Sharp, Gregory A. Light, Kara J. Blacker

**Affiliations:** ^1^ Warfighter Effectiveness Research Center, U.S. Air Force Academy, Colorado Springs, CO, United States; ^2^ Naval Medical Research Unit-Dayton, Wright-Patterson AFB, Dayton, OH, United States; ^3^ Department of Psychiatry, University of California San Diego, San Diego, CA, United States; ^4^ VISN-22 Mental Illness, Research Education and Clinical Center, VA San Diego Healthcare System, San Diego, CA, United States; ^5^ The Henry M. Jackson Foundation for the Advancement of Military Medicine, Inc., Bethesda, MD, United States

**Keywords:** hypoxia, event-related potentials, mismatch negativity, P3a, auditory processing

## Abstract

The ability to detect novelty in our environment is a critical sensory function. A reliable set of event-related potentials (ERP), known as the auditory deviance response (ADR), are elicited in the absence of directed attention and indexes functionally relevant networks. The ADR consists of three peaks: mismatch negativity (MMN), P3a, and reorienting negativity (RON) that are sequentially evoked in response to unattended changes in repetitive background stimulation. While previous studies have established the ADR’s sensitivity to a range of pharmacologic and nonpharmacologic interventions and are leading candidate biomarkers of perturbations of the central nervous system (CNS), here we sought to determine if ADR peaks are sensitive to decreases in breathable oxygen. Participants performed a visuomotor tracking task while EEG was recorded during two 27-min sessions. The two sessions differed in the amount of environmental oxygen available: 10.6% O_2_ (hypoxia) versus 20.4% O_2_ (normoxia). ERPs were measured while a series of identical, or “standard,” tones combined with occasional “oddball,” tones, were presented. MMN, P3a, and RON were assessed in response to the oddball compared to the standard stimuli. Behavioral impairment during hypoxia was demonstrated by a deficit in tracking performance compared to the normoxia condition. Whereas no changes were detected in the MMN or RON, the amplitude of the P3a component was significantly reduced during hypoxia compared to normoxia, within the first 9 min of exposure. To our knowledge, this is the first study to demonstrate the effect of low oxygen exposure on passively elicited neural measures of early sensory processing. This study demonstrates that passively elicited EEG measures, reflecting preattentive auditory processing, are disrupted by acute hypoxia. Results have implications for the development of biomarkers for the noninvasive assessment of CNS perturbations.

## Introduction

The amount of information present in the surrounding environment at any given moment far exceeds our perceptual and cognitive capacities. To cope with this surplus of sensory input, the central nervous system (CNS) seamlessly governs what information transitions from routine sensory processing to allow for the allocation of additional cognitive resources necessary for processing unexpected changes in the environment (1). The efficiency of this early information processing can be reliably assessed using event-related potentials (ERPs). The mismatch negativity (MMN), P3a, and reorienting negativity (RON) are ERP measures that are sequentially evoked as a triphasic auditory deviance response (ADR) complex [(2); but also see (3)], and can reliably index automatic and preattentive stages of early auditory information processing (4–6). The ADR is passively elicited when a series of frequently presented auditory tones (i.e., “standards”) that are identical in pitch and duration (e.g., “beep, beep, beep, beep…”) is occasionally interrupted by a physically deviant (i.e., “oddball”) tone (e.g., “beeeeep”). The ADR requires no overt behavioral response from the participant and can be measured in the absence of directed attention, usually while participants perform some other active or passive foreground task in the visual domain.

Notably, ADR responses can be measured in rodents, nonhuman primates, fetuses, sleeping infants and adults, and even comatose individuals (7–12). The MMN response peaks at 120-200ms and is followed by a positive-going waveform, the P3a, which peaks at 250–320 ms. In some cases, the P3a is followed by a negative going wave that peaks at 350–450 ms known as the RON (13–15). While the MMN represents a preattentive sensory discrimination process, the P3a is thought to reflect a higher-order orienting or covert shifting of attentional resources to an unexpected stimulus. RON is thought to reflect a reorienting of attention back to the foreground task, though the underlying psychological construct is less developed. Important for the current study, the ADR complex has been shown to be sensitive to a number of CNS perturbations including acute pharmacologic challenges and cognitive training interventions (16–22). The ADR components are translational biomarkers elicited in the absence of directed attention and index functionally relevant networks, thus they are increasingly used as biomarkers in the development of interventions that target CNS dysfunction (17, 21, 23–25).

Despite the automaticity of this novelty detection function, these processes carry a neural expenditure that requires energy, which is partially supplied through oxygen. While the average human brain represents only about 2% of one’s total body weight, it accounts for approximately 20% of the oxygen consumption for the entire body (26). Therefore, any decrement in the amount of breathable oxygen available may impact a host of neural functions, perhaps even the earliest sensory information processes, like those measured by the ADR complex.

The condition in which an organism has insufficient environmental oxygen is known as hypoxic hypoxia (differing from hypoxia brought about by other factors, such as histotoxic hypoxia). Notably, hypoxic hypoxia occurs in healthy individuals when they ascend to high altitude or when breathing mixtures of gases with low oxygen content (e.g., while underwater diving), particularly when using rebreather systems that control the amount of oxygen in the supplied air. For simplicity, subsequent use of the term “hypoxia” will indicate hypoxic hypoxia.

The symptoms of hypoxia depend on the severity (i.e., dose) and exposure time. In cases of altitude sickness in which hypoxia develops gradually, symptoms can include fatigue, numbness or tingling of extremities and nausea (27), but specific symptoms have been shown to be highly idiosyncratic in other settings (e.g., flight training; 28). Despite individual differences in some symptom reports, hypoxia in general has been shown to negatively affect performance on a range of perceptual, cognitive, and motor tasks. For example, hypoxia increases pupillary response latency (29), changes perception of stimulus intensity (30), impairs color vision (31), and reduces reaction time [(RT; (29, 32–34)]. Further, hypoxia often results in decreases in working memory (35, 36), as well as other higher-order cognitive tasks [(37); for a review see, (38)]. Given these documented impairments under hypoxic conditions, there is great interest in the aviation and diving communities to develop novel methods for assessing the brain’s response to hypoxia in otherwise healthy individuals. This endeavor could result in a more effective way to study hypoxia in the laboratory and/or as a tool to help train individuals to identify their hypoxia symptoms before performance is impaired.

Moreover, it has been established that the electrical activity of the brain is sensitive to its oxygen supply. Some studies have shown changes in resting state spectral EEG activity, such as increased delta and/or theta power, as well as changes in alpha power (depending on whether eyes were opened versus closed during recording) under hypoxic compared to normoxic conditions (39–42). While these studies demonstrate the sensitivity of resting state EEG measures to hypoxia, the reported changes in spectral power provide little insight into the specific underlying perceptual or cognitive functions that are affected.

Few studies to date have examined ERPs to investigate the influence of hypoxia on sensory and/or higher-order cognitive processing. The results of one early study of an active auditory target detection task showed evidence of a delayed latency of the P3b component to target stimuli that corresponded to a lag in RT to those auditory stimuli (32). More recently, Altbäcker et al. (43) assessed the sensitivity of three variants of the P300 component (i.e., Target P3, No Go P3, and Novelty P3) to hypoxia evoked in response to a modified continuous performance task. Similar to that found by Fowler and Lindeis (32), these components were elicited *via* an active target detection task while participants attended to a continuous stream of letters. The results demonstrated that the Novelty P3, but not the other components, was significantly decreased under hypoxic compared to normoxic conditions. These two previous studies suggest that the P300 component, when elicited in response to an active foreground cognitive task, is sensitive to hypoxia.

Given that (1) the brain requires a substantial amount of oxygen to function, (2) neuroelectrical activity is notably affected by hypoxia, and (3) deficits in sensory and higher-order cognitive processes are associated with hypoxia, it is reasonable to suspect that the lower levels of background sensory processing may also be impacted by an insufficient supply of oxygen. Thus, the current study sought to examine the sensitivity of the MMN, P3a, and RON components to acute normobaric hypoxia exposure. Since these ADR components are leading candidate biomarkers for predicting and monitoring response to interventions, determining the profile and time course of response to acute hypoxia exposure represents an important biomarker validation for future application. We anticipated that this signal complex would demonstrate a reduction in all peak amplitudes during hypoxic compared to normoxic conditions. Follow up analyses were also performed to assess the relative timing of this reduction in relation to oxygen saturation (SpO_2_) levels and performance on a foreground behavioral task.

## Materials and Methods

### Participants

A total of 40 healthy adults (age: *M* = 29.43, *SD* = 5.99; 27 males) participated for monetary compensation. All participants were recruited through flyers and online announcements. Participants who completed the study received $200. All participants enrolled in the study self-reported normal or corrected-to-normal vision, no history of psychological, neurological, or medical diagnosis, no use of tobacco in the past 6 months, and no excessive alcohol use. Participants were not administered a hearing test as part of the study, but all but two participants were active-duty military who receive baseline and periodic follow-up audiometry as part of their regular health assessment. Participants gave written informed consent approved by the Institutional Review Board of the Naval Medical Research Unit - Dayton.

### Experimental Design and Procedures

**Figure 1:**
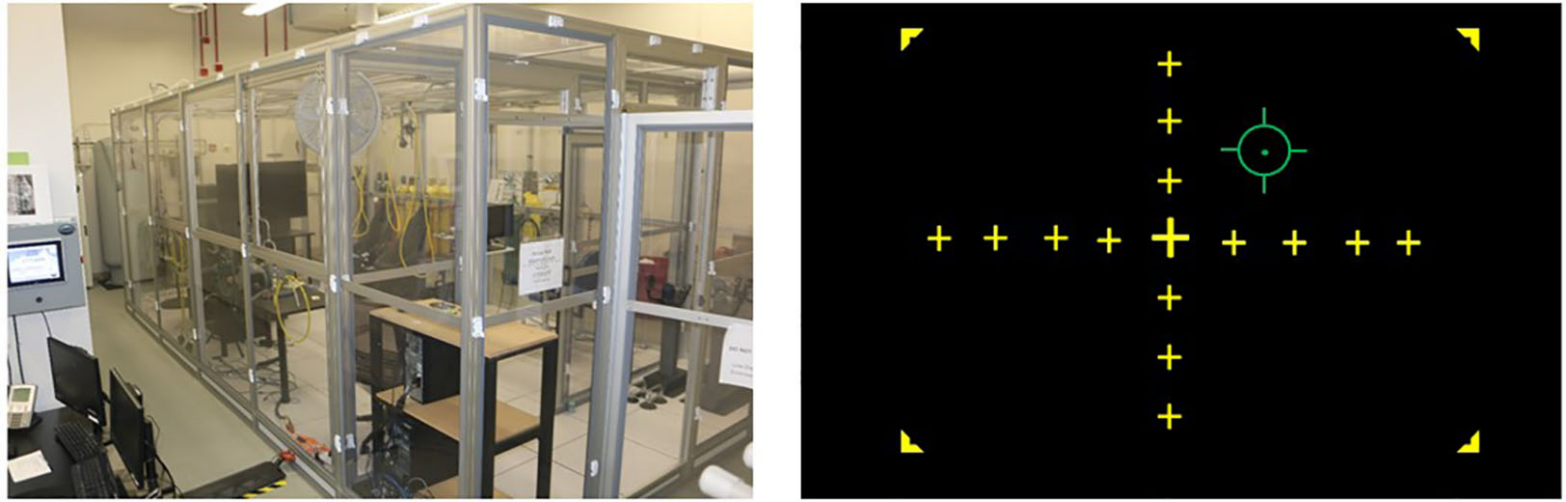
Left: Reduced Oxygen Breathing Environment (ROBE) where experimental sessions took place (Photo courtesy of Naval Medical Research Unit - Dayton). Right: Visuomotor tracking task schematic. Participants were tasked with using a joystick to keep the green reticle in the center of the yellow crosshairs over the course of a 27-min exposure.

Participants completed two counterbalanced experimental sessions separated by an average of 8.7 days (*SD* = 9.9). Testing was conducted in a Reduced Oxygen Breathing Environment (ROBE; **Figure 1**), which simulates the oxygen concentrations of high altitudes without the risks associated with reduced barometric pressure found in altitude chambers or other hypoxia replication devices. The two experimental sessions differed only in the oxygen content found within the testing chamber on the days of the participation. During the normoxia session, participants were exposed to near sea-level equivalent room air (20.4% O_2_), while during the hypoxia session, they were exposed to a 10.6% O_2_ mixture (i.e., environmental equivalent of ~17,500 ft). Throughout the experimental testing sessions, participants wore a NoninConnect Model 3230 finger-mounted pulse oximeter (Nonin Medical, Inc.) to index SpO_2_ and heart rate for both safety monitoring purposes and as variables of interest.

### Behavioral Tracking Task

Participants were seated approximately 90 cm from a 21” monitor and used a joystick to align an independently moving reticle (using a sinusoidal equation to provide pseudorandom motion requiring constant correction by the participant) with the center point of a crosshair displayed on the screen (see **Figure 1**). Performance on this task was assessed as the difference or error in pixels between the center of the reticle and the center of the crosshair display, which was recorded at a 10 Hz sampling rate. Prior to their first session, participants practiced the tracking task outside the ROBE for 5 min to acclimate to the sensitivity of the joystick.

### EEG Data Acquisition

EEG data were recorded continuously in the ROBE from 64 electrodes covering the whole scalp with approximately uniform density using an elastic electrode cap (ActiCHamp, Brain Products) referenced to the right mastoid (TP9) in DC mode, at a sampling rate of 1000Hz. Electrode impedance for all channels was kept below 10 kΩ.

Auditory stimuli were presented to participants at 85 dB sound pressure level *via* Etymotic ER3-A insert earphones. The passive auditory oddball paradigm used to elicit ADR measures comprised a sequence of tones, of which 85% were standards (50 ms, 1,000 Hz, *n* = 2754) and 15% were deviants (7.5%, *n* = 243 per deviant type) that differed in stimulus duration (100 ms, 1,000 Hz) or both duration and frequency (i.e., “double-deviant” 100 ms, 1,100 Hz). These two deviant types were selected based on previous work demonstrating that these conditions have excellent test-retest reliability, strong associations with cognition and daily functioning, and sensitivity to interventions (17, 20–22, 25, 44). All tones had 5 ms rise/fall times and were presented with a fixed 500 ms stimulus onset asynchrony. Participants were instructed to ignore auditory stimuli while they completed the visuomotor tracking task, described above.

### Statistical Analysis

Visuomotor tracking performance was measured as the amount of error (in pixels) between the reticle that the participant controlled and the stationary center target. Data were binned into three, 9 min bins per session. SpO_2_ and heart rate (beats per minute; BPM) data were also binned into three, 9 min bins and compared across time and session.

EEG data were processed using EEGLab and Brain Vision Analyzer. Data were down-sampled to 500 Hz and a 0.5-Hz high pass filter, and common average reference were applied for initial preprocessing. Independent Component Analysis (ICA) was applied to continuous data to minimize ocular artifacts. Data were segmented around the stimuli, −100 to 500 ms, baseline corrected, and screened for residual artifacts where segments with amplitudes exceeding ±100 μV in frontocentral electrodes (Fz, FCz, Cz) being rejected. ERP averages for standards and deviants (both types together) were generated separately, and the resultant difference waves were low-pass filtered at 20 Hz. Deviants were initially considered separately, but no difference was found between the two types and therefore were averaged together for all analyses. Waveforms were rereferenced to linked mastoids, with ADR amplitudes calculated for data across the entire 27 min recording as well as binned into three bins of 9 min. Statistical analyses were performed using SPSS v26 and focused on differences in the mean MMN (120–200 ms), P3a (250–320 ms), and RON (350–450 ms) time windows at an *a priori* defined electrode of interest (i.e., Fz) during hypoxia and normoxia over the three blocks of testing (each 9 min).

## Results

One participant was excluded from all analyses due to below chance level performance on the visuomotor tracking task during both experimental sessions. Three additional participants dropped below our safety designation (i.e., SpO_2_% < 60) during the hypoxia session and the session was terminated early. Those three participants’ data were included in the subsequent analyses where possible^1^. One participant’s performance data for the normoxia session was lost due to experimenter error. Four participants did not have heart rate data recorded.

### Behavioral Performance

**Figure 2:**
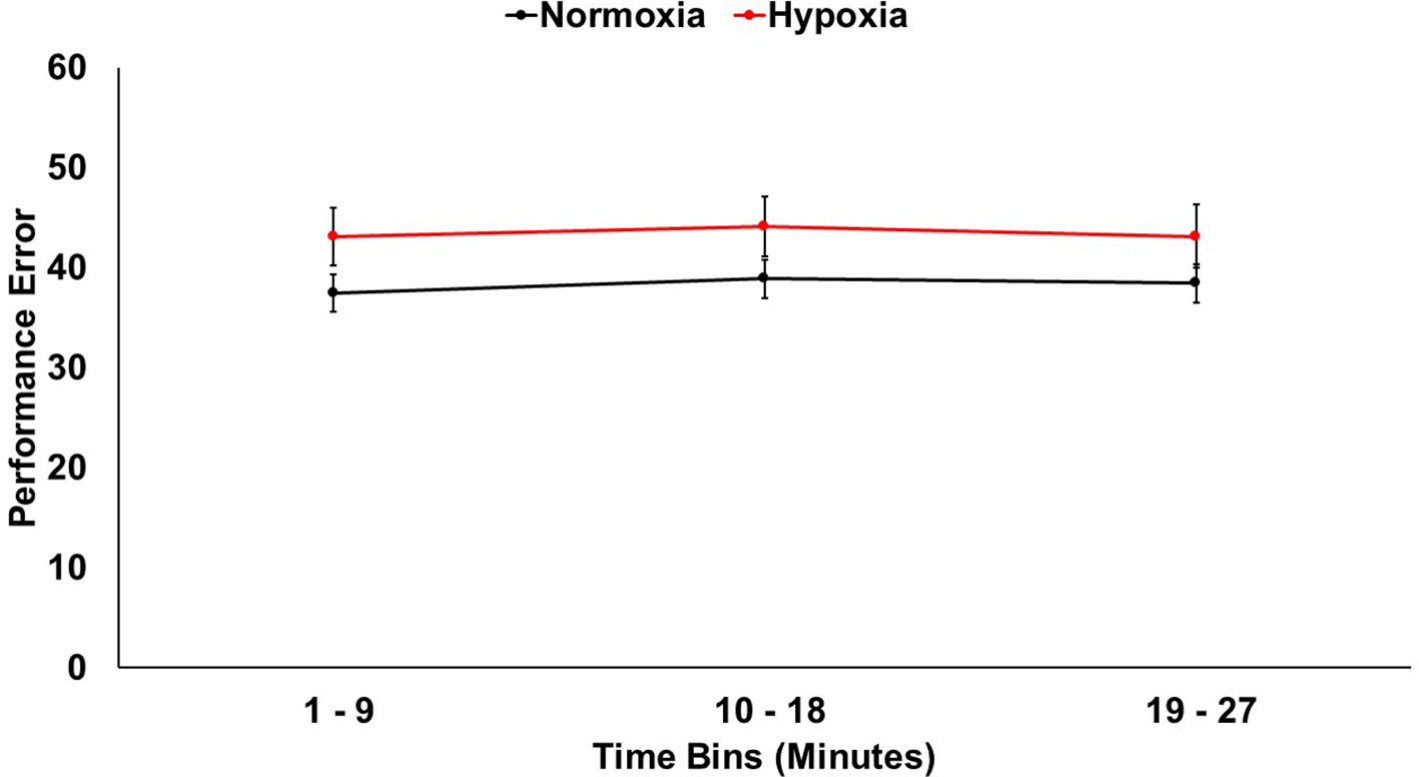
Visuomotor tracking task performance shown for hypoxia and normoxia sessions separately. Performance was significantly worse (more error) for hypoxia compared to normoxia. Error bars represent standard error of the mean.

A 2 (session: normoxia vs. hypoxia) × 3 (time bin) repeated-measures ANOVA was tested on error (**Figure 2**). The main effect of time was not significant, *F*(2,33) = 1.08, *p* = 0.34. The main effect of session was significant, *F*(1,34) = 6.07, *p* < 0.05, *η_p_^2^* = 0.15, with performance being impaired in the hypoxia session compared to the normoxia session. The session x time interaction did not reach significance, *F*(2,33) = 0.24, *p* = 0.79.

### Physiological Monitoring

**Figure 3:**
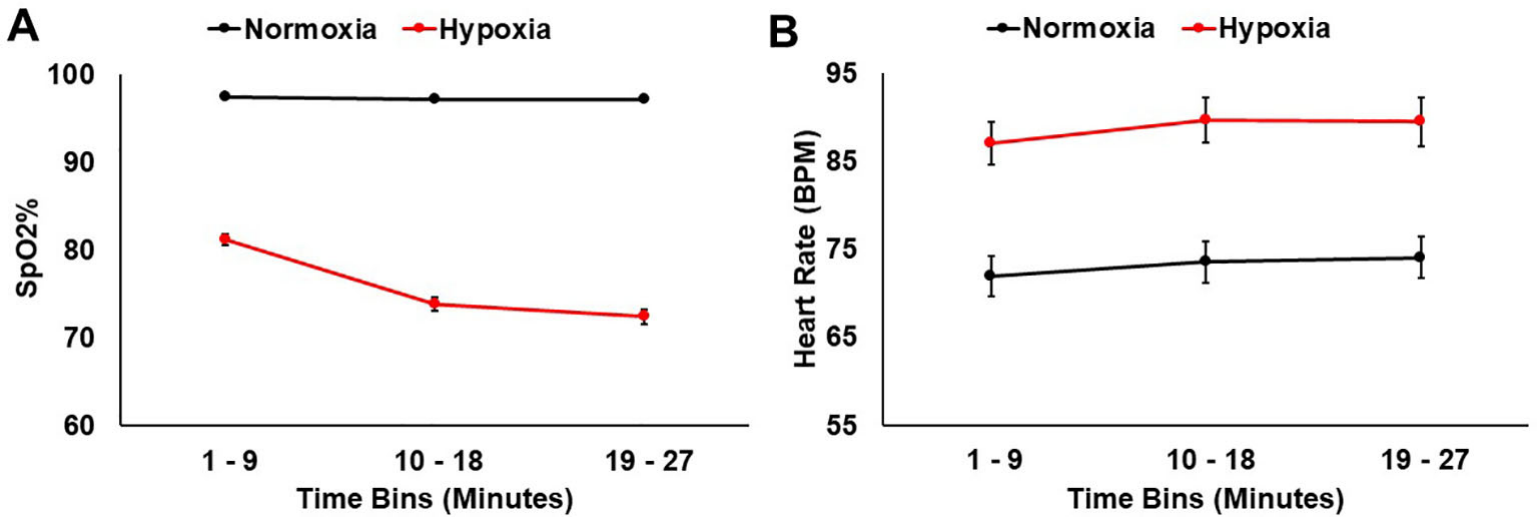
**(A)** SpO_2_% and **(B)** heart rate data shown for hypoxia and normoxia sessions separately. Error bars represent standard error of the mean.

SpO_2_ and heart rate were monitored throughout the 27 min for both sessions. A 2 (session: normoxia vs. hypoxia) × 3 (time bin) repeated-measures ANOVA was tested on SpO_2_%. Both the main effect of session, *F*(1,36) = 691.36, *p* < 0.001, *η_p_^2^ =* 0.95, and the main effect of time bin, *F*(2,35) = 157.18, *p* < 0.001, *η_p_^2^ =* 0.81, were significant with lower SpO_2_% during hypoxia and in the later time bins. The session x time bin interaction, *F*(2,35) = 128.12, *p* < 0.001, *η_p_^2^ =* 0.78, was also significant, demonstrating that SpO_2_% dropped over time more drastically during hypoxia compared to normoxia (**Figure 3A**).

A 2 (session: normoxia vs. hypoxia) × 3 (time bin) repeated-measures ANOVA was also tested on heart rate. Both the main effect of session, *F*(1,32) = 83.84, *p* < 0.001, *η_p_^2^ =* 0.72, and main effect of time bin, *F*(2,31) = 14.19, *p* < 0.001, *η_p_^2^ =* 0.31, were significant with heart rate being higher in the hypoxia session and in later time bins (**Figure 3B**). The session x time interaction did not reach significance, *F*(2,31) = 0.55, *p* = 0.55.

### Assessment of Hypoxia Effects on MMN, P3a, and RON Amplitudes

**Figure 4:**
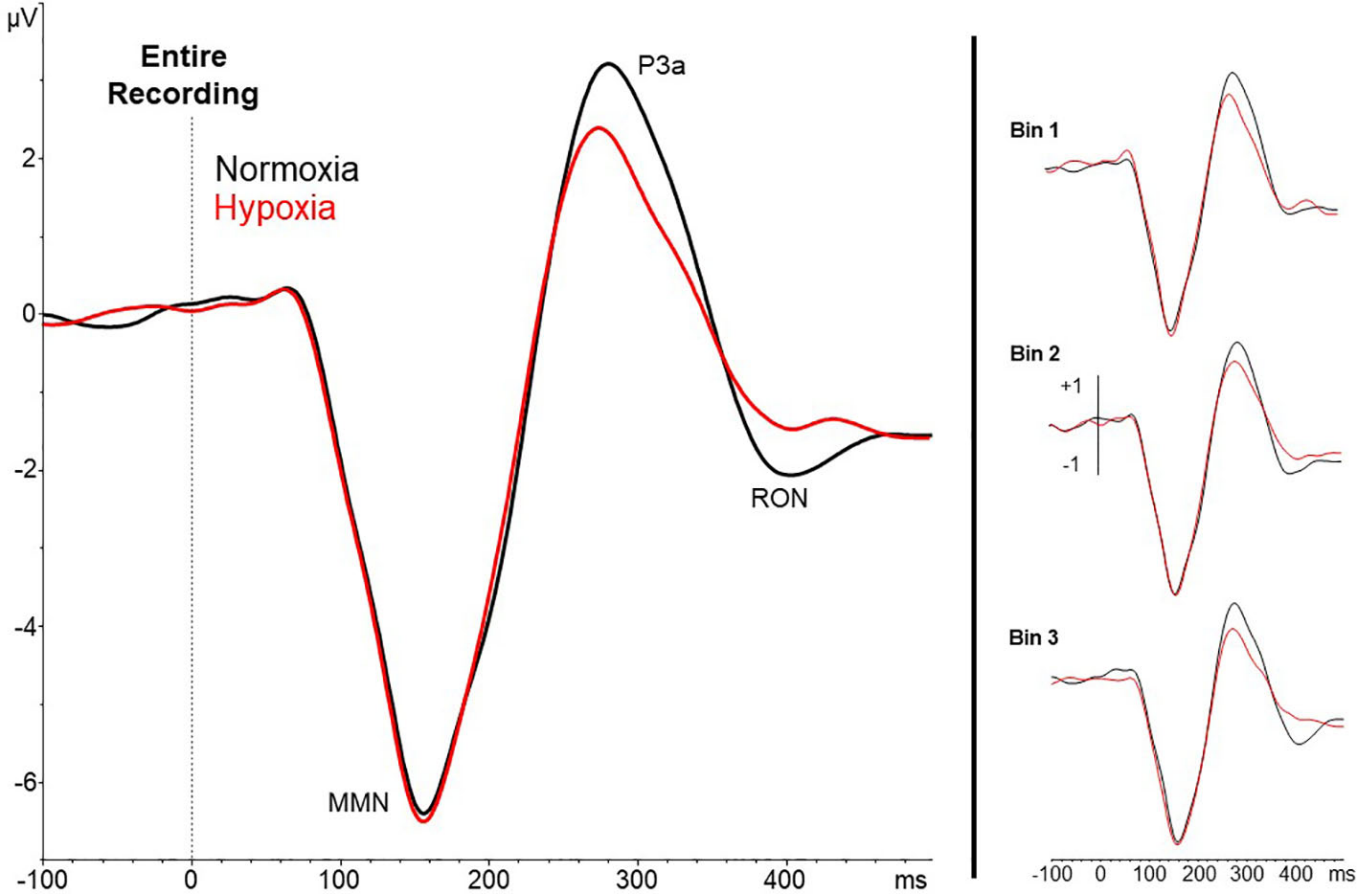
Grand averaged waveforms (deviant – standard) from electrode Fz for the entire 27-min exposure (left) and the three 9-min time bins (right). Results show a reduced amplitude P3a under hypoxic compared to normoxic conditions.

**Figure 5:**
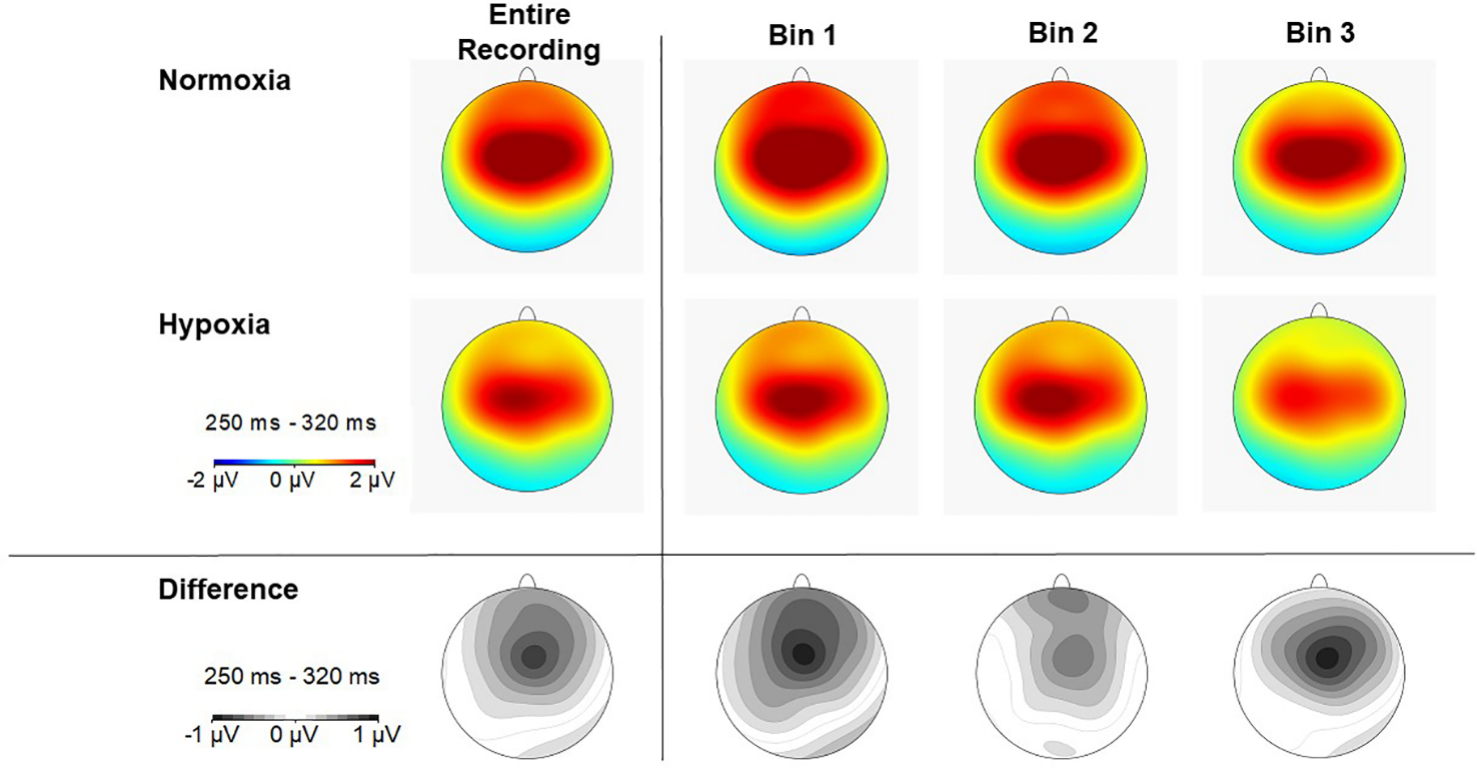
Scalp topography of P3a amplitude (250–320ms) in each time bin for normoxia (top row), hypoxia (middle row), and the difference between conditions (bottom row).

A 2 (session: normoxia vs. hypoxia) x 3 (time bins) repeated-measures ANOVA was tested separately for MMN, P3a, and RON amplitudes. For the MMN, neither the main effect of time, *F*(2,35) = 0.71, *p* = 0.50, nor session, *F*(1,36) = 0.06, *p* = 0.81, reached significance. The time x session interaction was also nonsignificant, *F*(2,35) = 0.23, *p* = 0.80. For the P3a, significant main effects of session, *F*(1,36) = 18.6, *p* = 0.0001, *η_p_^2^ =* 0.34, and time bin, *F*(2,35) = 5.92, *p* < 0.01, *η_p_^2^ =* 0.25, were observed with reduced P3a amplitude during hypoxia and over time (**Figures 4** and **5**). The session x time bin interaction did not reach significance, *F*(2,35) = 2.14, *p* = 0.13. For the RON, the main effect of time was significant, *F*(2,35) = 4.54, *p* = 0.02, *η_p_^2^ =* 0.19, but neither the main effect of session, *F*(1,36) = 0.01, *p* = 0.94, nor the interaction, *F*(2,35) = 1.28, *p* = 0.29, approached significance.

## Discussion

The results of the present study demonstrate that passively elicited EEG measures, reflecting preattentive auditory information processing, are disrupted by acute hypoxia exposure. To our knowledge, no previous work has demonstrated the effect of low oxygen exposure on passively elicited neural measures of sensory processing. Specifically, using a passive auditory oddball paradigm in conjunction with a continuous visuomotor tracking task, a significant reduction in P3a amplitude during a hypoxic compared to a normoxic condition was observed. Importantly, this reduction was evident within the first 9 min of the exposure and corresponded to a significant impairment in concurrent performance on a foreground behavioral task.

Previous work involving the P300 signal has shown that the P3b component, when *actively* elicited in response to a foreground target detection task, is sensitive to hypoxia exposure (32, 43). Whereas these prior studies have examined the P3b subcomponent in response to correctly identified task-relevant target stimuli (and required an active response on the part of the participant), the present data represent the first report showing that even *passively* elicited P3a in response to background, task-irrelevant, sensory stimuli is also disrupted by hypoxia. The finding that P3a is acutely sensitive to a nonpharmacologic CNS perturbation has implications for the use of this and related measures of early auditory information processing as biomarkers in clinical trial designs [e.g., (25, 45, 46)]. Interestingly, no changes in the earlier MMN or later RON components were detected. Future studies are needed to confirm the specificity of P3a in response to hypoxia exposure.

Low oxygen conditions represent a risk to any exposed individual due to the known impact on a variety of behavioral performance outcomes, including RT (29, 32–34), perceptual sensitivity (30, 31), and higher-order cognitive functions [for a review see, (38)]. One particular environment where hypoxia is an ever-present threat is in tactical aviation. Current military aircraft are not equipped with a failsafe warning system to detect conditions that cause or exacerbate hypoxia. Instead, operators must recognize a broad range of idiosyncratic hypoxia symptoms, such as tingling in extremities, light-headedness, difficulty concentrating, and slowed RTs before they become incapacitated (28), which has been compared to the reliability of an intoxicated individual determining if he/she is safe to drive. Ancillary to these subjective symptoms, exposure to reduced levels of breathable oxygen has been documented to affect an operator’s ability to maintain a constant airspeed, altitude, and directional heading during simulated flights (37, 47). Thus, the establishment of a neural biomarker of hypoxia using the P3a would benefit research into the onset and effects of hypoxia on performance measures relevant to tactical aviation.

Here, we observed that the P3a amplitude reduction occurred in conjunction with concurrent behavioral performance. One limitation to the current study is the resolution at which we could examine the ERP effects. It is possible that the P3a reduction occurs prior to this performance deficit, but in the current study we lacked adequate resolution to detect that effect. Future studies should aim to reduce the acquisition time needed to get a reliable P3a, in order to examine the predictive potential of the component during low oxygen exposure. Novel analytic approaches may allow for a more rapid detection of changes in EEG dynamics [e.g., (48)]. Alternatively, since scalp-level ERPs represent a variable mixture of contributions from multiple brain regions, examination of source-level brain dynamics may allow for a more rapid detection of changes secondary to hypoxia or other CNS perturbations [e.g., (20)]. A second limitation was the use of only one altitude for the hypoxia manipulation. The current results represent a first step and future studies should examine whether the reduction in the amplitude of the P3a changes with lower levels of breathable oxygen and presumably more impaired performance.

In conclusion, the current study showed for the first time that a passively elicited neural measure of sensory processing, the P3a, is sensitive to acute hypoxia exposure. The reduction in P3a amplitude during a 10.6% O_2_ exposure represents a first step in exploring the utility of the P3a as a biomarker of hypoxia-related insults.

## Data Availability Statement

The datasets generated for this study are available on request to the corresponding author.

## Ethics Statement

The studies involving human participants were reviewed and approved by Naval Medical Research Unit - Dayton IRB. The patients/participants provided their written informed consent to participate in this study.

## Author Contributions

TS and GL generated the idea for the study. RS programmed the auditory oddball stimuli. TS, GL, and KB designed the experiment. TS and KB collected the data. MF, GL, RS, and KB analyzed the data. KB wrote the first draft of the manuscript and TS, MF, and GL critically edited it. All authors approved the final submitted version of the manuscript.

## Funding

This work was supported by Office of Naval Research award 0601153N to Naval Medical Research Unit-Dayton, with Principal Investigator TS, U.S. Navy.

## Disclaimer

The views expressed in this article reflect the results of research conducted by the authors and do not necessarily reflect the official policy or position of the Department of the Navy, Department of Defense, nor the United States Government.

## Conflict of Interest

The authors declare that the research was conducted in the absence of any commercial or financial relationships that could be construed as a potential conflict of interest.

## Footnotes

1 All analyses were tested with and without these three participants included. Excluding them did not change the direction or significance of any reported effects.
